# Eleven Weeks of Iron Supplementation Does Not Maintain Iron Status for an Entire Competitive Season in Elite Female Volleyball Players: A Follow-Up Study

**DOI:** 10.3390/nu10101526

**Published:** 2018-10-17

**Authors:** Juan Mielgo-Ayuso, Michael C. Zourdos, Julio Calleja-González, Alfredo Córdova, Diego Fernandez-Lázaro, Alberto Caballero-García

**Affiliations:** 1 Department of Biochemistry, Molecular Biology and Physiology, Faculty of Physical Therapy, University of Valladolid, 42004 Soria, Spain; a.cordova@bio.uva.es; 2 Department of Exercise Science and Health Promotion, Muscle Physiology Laboratory, Florida Atlantic University, Boca Raton, FL 33431, USA; mzourdos@fau.edu; 3 Department of Physical Education and Sports, University of Basque Country (UPV-EHU), 1007 Vitoria, Spain; julio.calleja.gonzalez@gmail.com; 4 Department of Cell Biology, Histology and Pharmacology, Faculty of Physical Therapy, University of Valladolid, 42004 Soria, Spain; diego.fernandez.lazaro@uva.es; 5 Department of Anatomy, Faculty of Physical Therapy, University of Valladolid, 42004 Soria, Spain; director@iecscyl.com

**Keywords:** iron supplementation, exercise, female, health, volleyball eleven weeks of iron supplementation does not maintain iron status for an entire competitive

## Abstract

Background: Even though iron supplementation can be effective, it is necessary to be cautious of toxicity and aim to do no harm, therefore, it is important to examine the length of time the benefits of iron supplementation can be maintained following its cessation. The main purpose of this study was to analyze if iron stores and strength performance were maintained in elite female volleyball players for the final 18 weeks of a competitive season following the cessation of 11 weeks of iron supplementation. Methods: Twenty-two volleyballers (age: 27.0 ± 5.6 years.) were assigned to two groups (iron treatment group-ITG, *n* = 11 or control gropu-CG, *n* = 11) at the beginning of a previous trial (T0) and ITG consumed 325mg/d of ferrous sulphate for 11 weeks (T11). Then, in the present study iron status and strength were measured again 10 (T21) and 18 weeks later (T29) after the cessation of supplementation. Results: At the end of the previous trial (T11), ITG maintained iron status as measured by hematological parameters (serum iron-sFE, serum ferritin-FER, transferrin saturation index-TSI, and hemogloblin-Hb), however, CG showed a decrease in these markers at T11. Further, from T0 to T11 ITG experienced greater (*p* < 0.05) changes in clean and jerk, power clean, and total mean strength (TMS-sum of all strength tests) than CG. In the present, follow-up investigation, there was a group-by-time interaction in favor of CG vs. ITG from T11 to T21 for FER (*p* = 0.028) and Hb (*p* = 0.042). Further, there was an increase for CG (*p* < 0.001) in power clean for CG from T11 (38.4 ± 1.7 kg) to T21 (41.3 ± 1.9 kg) and T29 (41.8 ± 1.7 kg), but no change for power clean in ITG (*p* > 0.05). A group-by-time interaction from T11 to T29 occurred in favor of CG for half-squat (*p* = 0.049) and TMS (*p* = 0.049). Conclusion: Our findings suggest that the benefits of iron supplementation are not sustained in elite female volleyballers if supplementation is ceased for 18 weeks.

## 1. Introduction

The micronutrient iron aids athletic performance via enhancing oxygen transport capacity [1]. Consequently, elite athletes, due to increased mechanical hemolysis, gastrointestinal issues, and iron loss through sweat [2] are at an increased risk to for iron depletion, iron deficiency (ID) [3,4], and possibly iron-deficit anemia [5,6,7]. Indeed, diminished iron storage has been shown to negatively affect aerobic capacity, strength, muscular fatigue, and has delayed skeletal muscle recovery in elite athletes [8,9]. Due to the importance of maintaining iron stores, athletes might consider iron supplementation as ID can still occur even when the recommended dietary allowance (RDA) for iron is met [4]. Female athletes in particular should monitor their iron hematological profile [10]. Indeed, data have shown iron supplementation to prevent a decline in female athletes’ iron stores [9]. Further, a recent meta-analysis [8] and review [11] suggests that iron supplementation is effective to prevent or treat ID in female athletes.

In terms of specific sports, volleyball has been shown to negatively affect iron metabolism in females [4]. Indeed, previous work from our laboratory, Mielgo-Ayuso et al. (2015) [12], demonstrated 325 mg/day of ferrous sulphate (i.e., 105 mg/day elemental iron), to prevent a decline in iron stores and enhance strength in elite female volleyball players during 11 weeks of the competitive season compared to a control group [9]. Even though iron supplementation can be effective, it’s necessary to be cautious of toxicity and aim to do no harm [13], therefore, it is important to examine the length of time the benefits of iron supplementation can be maintained following its cessation. Newhouse et al. (1989) [14] suggested that in females with ID supplementation should span 16 weeks to reach recommended levels, however, it is not known how well these levels can be maintained without supplementation, especially in the presence of demanding training. If iron stores and performance can be maintained for a lengthy period following supplementation cessation, then more specific recommendations can be given to coaches and athletes regarding how long a supplementation protocol needs to last during a competitive volleyball season.

Therefore, the main purpose of this study was to follow-up on the previous 11 weeks trial [9] and analyze the hematological profile (i.e., serum iron-sFe, ferritin-FER, transferrin saturation index-TSI, transferrin-TRF, hemoglobin-Hb, and hematocrit-Hct) and strength levels in the next 18 weeks after supplementation (325 mg/day ferrous sulphate vs. control) ceased. It was hypothesized that after the 18-week follow-up the athletes who received supplementation would sustain greater iron status, as measured by the hematological profile and strength performance compared to a control group who did not have supplementation.

## 2. Materials and methods

### 2.1. Brief Description of the Previous Supplementation Period 

The present investigation is a follow-up to a previously published study [9]. The previous investigation monitored hematological markers and strength performance over 11 weeks of either 325 mg/day of ferrous sulphate supplementation (Ferro-gradumet^®^ Teofarma Srl. Pavia–Italia) (iron treatment group-ITG, *n* = 11) or no supplementation (control group-CG, *n* = 11) in 22 elite female volleyball players during the competitive season. The previous results [9] showed that CG experienced a significant decrease (*p* < 0.05) for sFe (−33.9%), FER (−34.6%), TSI (−35.3%), and Hb levels (−7.44%), however ITG experienced no hematological changes (*p* > 0.05). Furthermore, there was a greater (*p* < 0.05) percentage increase in clean and jerk (CG: +5.1 ± 20.9% vs. ITG: +29.0 ± 21.3%), power clean (CG: −5.8 ± 30.3% vs. ITG: +44.6 ± 56.6%), and total mean strength (TMS-sum of all strength tests) (CG: +10.9 ± 3.2% vs. ITG: +26.2 ± 3.6%) in ITG compared to CG. Thus, the previous results showed greater iron status and strength in ITG vs. CG after 11 weeks.

### 2.2. Study Design and Participants

**Figure 1:**
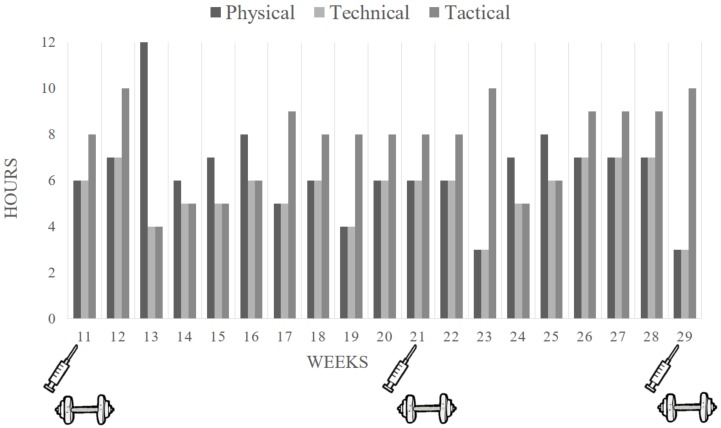
Time and type of training performed by volleyball players in each week of study.

The present investigation was conducted during the 18 weeks immediately following the initial 11-week period with the same 22 elite female volleyball players. All athletes performed the same training program, which was prescribed by the club’s coaches (Figure 1). Further, the club’s dietitian prescribed an individual diet for each player. The diets were prescribed using previously established energy and macronutrient guidelines for adequate athletic performance [15], and were based on the volume and training load, and personal characteristics of each participant. Importantly, diets were prescribed so that all athletes would meet the micronutrients Recommended Dietary Allowance (RDAs) for women aged 19–30 [16]. Importantly, the dietitian also aimed to avoid any interference of iron absorption. Specifically, iron-rich foods were not prescribed for consumption with foods, such as phytic acid, polyphenols, calcium, and peptides from partially digested proteins, which inhibit iron absorption. However, iron-rich were consumed along with foods such as ascorbate, citrate, some amino acids, and poultry and fish, which favor the absorption of iron [17].

Participants were determined to be free of disease by a medical examination and were without serious injury for 6 months prior to the study. Further, only female volleyball players who reported having a regular menstrual cycle were included. Additionally, no participants were using illegal drugs or taking medications, which affected body mass. The beginning and end of each menstrual cycle was noted during the study, since variations in iron status can occur during a cycle [18]. The experimental procedures, associated risks, and benefits were explained to all players and coaches. Each player signed a written consent form before participation. The study was designed according to Declaration of Helsinki (2008) and Fortaleza 2013, and approved by the Basque Country University (UPV-EHU) committee (CEISH/202R/2012/Mielgo Ayuso).

Iron status as measured by: sFe, FER, transferrin saturation index TSI, TRF, Hb, and Hct and participants were assigned at T0 (the beginning of the initial 11 weeks from the previous trial) based upon iron status and strength levels into either CG (*n* = 11, height: 179 ± 10 cm and body mass: 70.2 ± 7.9 kg; BMI: 22.0 ± 1.3 kg/m^2^), which did not consume iron supplement or 2. ITG (*n* = 11, height: 180±7cm and body mass: 69.5 ± 7.3 kg; BMI: 21.6 ± 2.0 kg/m^2^). For the present investigation participants reported to the laboratory for blood collection and strength testing at three specific points, which coincided with the periods during the season in which players would potentially be in peak performance for competition: 1. In December, at the end of the 11-week iron supplementation protocol (T11), 2. In March, 10 weeks after T11, coinciding with the Queen’s Cup competition, (T21); and 3. In April, during the final playoff matches, 8 weeks following T21 (T29).

### 2.3. Iron Status Categorization

Athletes were determined to be in one of four iron storage categories: (1) ‘Adequate iron stores’, (2) ‘Absolute iron deficiency’, (3) ‘Functional iron deficiency’ and (4) ‘Anemia’. The following criteria was used to categorize participants; Adequate iron stores: FER > 100 μg/L plus TSI > 20% and Hb > 12 g·dL^−1^, Functional iron deficiency: FER 30–99 μg/L or 100–299 μg/L plus TSI < 20% [19], Absolute iron deficiency: FER < 30 μg/L, and Anemia: Using an amount of Hb <12 g·dL^−1^ (women) [20].

### 2.4. Blood Collection and Analysis

Antecubital venous blood samples were collected from all players at T11, T21, and T29. All samples were collected in basal conditions after overnight fasting and at least 36 h following the last practice or match to avoid acute effects of exercise on blood markers. The players arrived at the laboratory at 8:30 a.m. and after 30 min of quiet-sitting, blood samples were collected. Further, no players were menstruating during blood collection time points.

Specifically, a blood sample was collected from the antecubital vein into a K-EDTA-coated Vacutainer system (4 mL) containing gel and clot activator for serum separation (9 mL). An STKS autoanalyzer (Coulter) was used to determine red blood cells (RBCs) and Hb, and Hct were also calculated. Additionally, the iron parameters SFe, TRF, and FER were measured using an auto-analyzer (COBAS FARA; Roche Diagnostics, Basel, Switzerland). Serum iron was determined using the colorimetric method with ferrozine, but without protein precipitation, while FER was measured via immunoturbidimetry. Lastly, TSI was calculated as: (TS (%) = SFe (mg/dL) × 70.9/TRF (mg/dL)) [21].

### 2.5. Strength Performance Testing

To evaluate strength performance, a battery of tests previously used in elite female volleyball players [9] were administered at each time point in an indoor sports hall with standard conditions (temperature: 21 °C and humidity: 60%). Tests were performed after a 20 min standardized warm-up (10 min of jogging, 5 min of jumping jacks, and 5 min of jumping rope, as well as accelerations and injury prevention drills).

During each testing session players performed submaximal strength tests of a 7–10 repetition maximum (RM) to examine pre-to post-testing changes in strength. The strength tests were as follows: Bench press, military press, back squat, power clean, clean and jerk, pull over, and TMS.

### 2.6. Dietary Assessment

The same dietitian who prescribed the diets instructed all participants on how to properly track food intake using two methods of dietary recall so that athletes could comply with the diets. The first method was to complete a validated food frequency questionnaire (FFQ) [22] following blood collection at each time point. This questionnaire has also been used in a similar population [23]. The FFQ, asked the participants to recall their average intake based on certain ‘frequency’ categories between time points from T11 to T29; the FFQ included 139 different foods and drinks, which were arranged by food type and meal pattern. Frequency categories were based on the number of times, where an item was consumed per day, per week, or per month. Daily consumption of energy (kcal) and each macro and micronutrient were determined by dividing the reported intake by the frequency in days [22].

The second method was for athletes to complete a 7-day dietary recall at T21 and T29 for the previous 7 days, to examine if results of this recall were like the FFQ. If participants had weighed food, then that data was used for the recall, however, if weighing food was not possible serving sizes consumed were estimated from the standard weight of food items or by determining portion size via looking at a book with 500 photographs of foods. Food values from FFQ were then converted into intakes of total energy, macro, and micronutrients including iron by a validated software package (Easy diet©, online version developed by the Spanish Centre for Higher Studies in Nutrition and Dietetics (CESNID), which is based on Spanish tables of food composition [24].

### 2.7. Statistical Analyses

Data are presented as means and standard errors, along with total ranges. Frequency and percentage were calculated and Pearson´s χ^2^ test was performed to calculate the differences in iron stores in CG and ITG between different points of study. Moreover, a Kruskal Wallis test was used to calculate the differences in iron stores in each time point between CG and ITG. Previously, the Shapiro-Wilk test was used to determine normality of data (*n* < 50), therefore we used parametric statistics. A Levene’s test was applied to measure the homoscedasticity of the variances. A repeated measure analysis of variance (ANOVA) was used to examine interactions (time × group) between ITG and CG for hematological and strength (for both kilograms and percentage change) parameters. A Bonferroni post-hoc test was applied for pairwise comparisons. Further, to analyze which Δ hematological parameters were the best predictors of Δ strength, a stepwise regression model was used with Δ strength performance tests (one to one) as the dependent variables and the Δ hematological parameters as predictors. Additionally, group differences at the end of the 11-week iron supplementation protocol (T11) and the percentage change of the outcome variables from T11 to T29 were calculated as Δ (%): ((T29 − T11)/T11) × 100. Differences from T0 to T11 were assessed by a non-paired Student’s t test or Mann–Whitney U-test, after normality of the data had been confirmed with the Shapiro-Wilk test, to decide parametric or non-parametric analysis. Statistical significance was indicated at *p* < 0.05. Statistical analyses were performed using the IBM Statistical Package (SPSS Version 24, IBM Corp., Armonk, NY, USA) and Graphpad Prism (Graphpad Software Version 6. San Diego, CA, USA).

## 3. Results

### 3.1. Haematological Parameters

**Figure 2:**
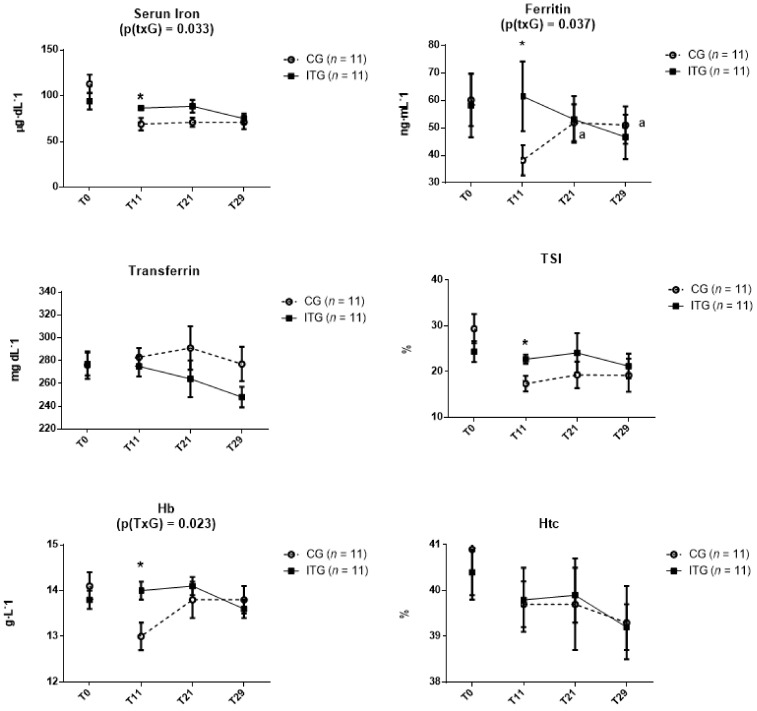
Hematological parameters in control group (CG) and iron treatment group (ITG) during follow up study. Data are expressed as mean and standard error. p(TxG) indicates interaction between treatment and time. * Significant differences between groups. a: Significant differences from T11.

At T11, there were significantly greater levels of sFe, FER, TSI, and Hb in ITG vs. CG (*p* < 0.05), while no difference existed between groups for TRF and Hct (*p* > 0.05), previously described by Mielgo-Ayuso et al. [9]. There was no group difference (*p* > 0.05) at T21 or T29 for any hematological variable. However, there was a significantly greater increase for CG compared to ITG from T11 to T21 for FER (*p* = 0.028) and Hb (*p* = 0.042). Additionally, in CG there was a significant increase in FER from T11 (38.2 ± 5.5 ng mL^−1^) to T21 (51.9 ± 6.7 ng mL^−1^; *p* = 0.028) and from T11 to T29 (51.0 ± 6.8 ng mL^−1^; *p* = 0.04) (Figure 2). Additionally, there were no differences for any hematological parameter between T0 and T29 for either group.

### 3.2. Iron Status Categorization

Specifics of iron categorization are displayed in Table 1. There was a significant difference between groups at T11 (*p* = 0.046), showing better iron stores in ITG vs. CG. However, at T21 and T29 there were no group differences (*p* > 0.05). In CG iron stores did not change (*p* > 0.05) from T11 to T21 or from T21 to T29. In ITG, iron stores did not change from T11 to T21 (*p* > 0.05), however, iron stores in ITG significantly decreased from T21 and T29 (*p* = 0.017). Specifically, in ITG, three additional athletes were categorized as having absolute iron deficiency at T29 compared to T11.

### 3.3. Strength Performance Tests 

**Figure 3:**
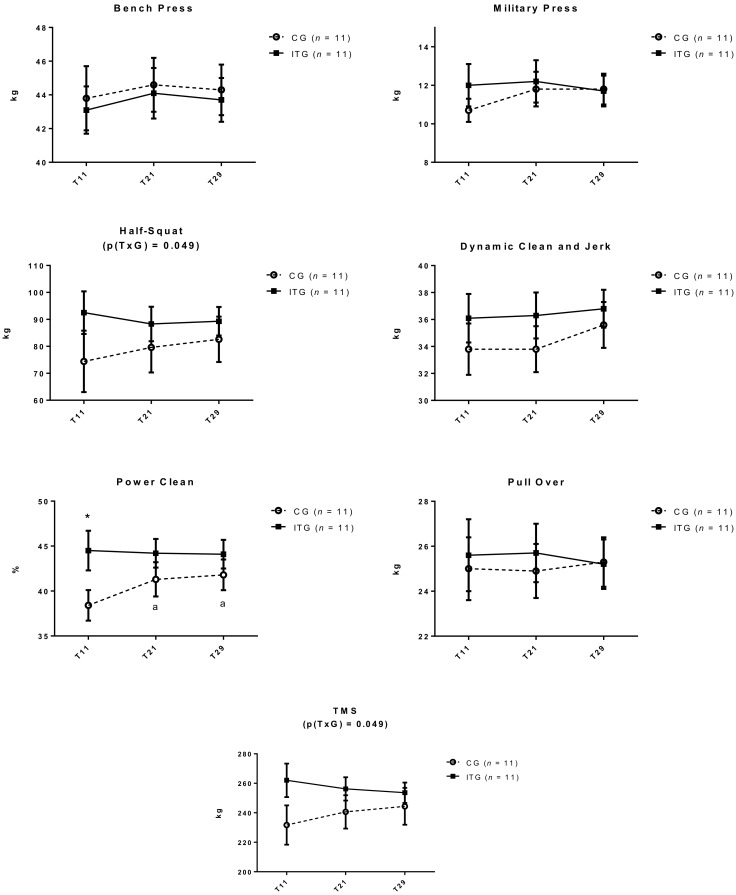
Changes in Strength Performance Tests in control group (CG) and iron treatment group (ITG) at T11, T21, and T29. Data are expressed as mean and standard error. TMS: Sum of all strength tests divided by 6. p(TxG) indicates interaction between treatment and time. * Significant differences between groups.

Figure 3, displays changes in all strength parameters at T11, T21, and T29. At T11 there were no significant differences (*p* > 0.05) between groups for any strength measure, except for power clean, which was greater in ITG (*p* < 0.05).

In terms of significant changes in strength, the only significant findings were an increase in power clean in CG (*p* < 0.001) from T11 (38.4 ± 1.7 kg) to T21 (41.3 ± 1.9 kg) and was sustained at T29 (41.8 ± 1.7 kg), and a group-by-time interaction from T11 to T29 in favor of CG for half-squat (*p* = 0.049) and TMS (*p* = 0.049).

### 3.4. Percentage Changes Differences for Hematological and Strength Parameters.

**Figure 4:**
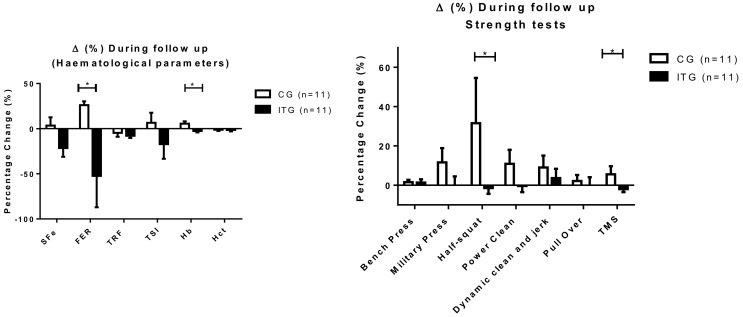
Percentage changes during follow up on hematological parameters and strength performance tests in control group (CG) and iron treatment group (ITG). Data are expressed as means ± standard error. Δ (%): ((T29 − T11)/T11) × 100; FER= serum ferritin; Hb = hemoglobin; Hct = hematocrit; sFe = serum iron; TSI = transferrin saturation index; TMS = Total Mean Strength: Sum of all strength tests divided by 6. * Significantly different between groups, *p* < 0.05.

Despite there being no group differences in terms of means at T21 and T29 for hematological or strength parameters, the ANOVA did detect significant interactions regarding percentage change (Figure 4). Specifically, there were significantly greater increases in favor of CG compared to ITG from T11 to T29 for FER (CG: +26.1 ± 4.2% vs. ITG: −52.2 ± 34.8%; *p* = 0.007), Hb (CG: +5.5 ± 2.6% vs. ITG: −2.5 ± 1.7%; *p* = 0.015) half-squat (CG: +31.5 ± 23.1 vs. ITG: −1.4 ± 3.0%; *p* = 0.036) and TMS (CG: +5.6 ± 4.1% vs. ITG: −1.9 ± 1.5%; *p* = 0.049).

### 3.5. Relationship Between Hematological and Strength Changes

Table 2 displays a multivariate regression with hematological changes (SFe, FER, TSI, TRF, and Hb) as the independent variables and percentage changes in strength as the dependent variables. There were significant relationships between changes in FER and TRF from T11 to T29 (*p* < 0.05) with certain strength measures; however, no other relationships existed (*p* > 0.05). Specifically, ΔFER was significantly associated with change in pull over strength (y = 3.549 + 0.123 × ΔFER; *p* = 0.004; *R*^2^ = 0.580), while ΔTRF was associated with back squat strength (y = −8.588−0.967 × ΔTRF; *p* = 0.017; *R*^2^ = 0.427). Finally, TRF (y = −2.735−0.501 × ΔTRF; *p* = 0.008; *R*^2^ = 0.689) and FER (y = −2.735−0.501 × ΔFER; *p* = 0.010; *R*^2^ = 0.689) were both associated with changes in ΔTMS.

### 3.6. Dietary Intake

Table 3 displays mean energy (kcal), macronutrient, and micronutrient intake throughout the entire 18 weeks, along with showing the RDA for each micronutrient [16]. There was no significant difference between groups for total kcal intake or intake of any macro or micronutrient (*p* > 0.05). Additionally, both groups met the micronutrient RDAs for women aged 19–30.

## 4. Discussion

The primary aim of this study was to examine iron status (SFe, FER, TSI, TRF, Hb, and Hct) and strength performance in elite female volleyball players during a season in the 18 weeks immediately following cessation of 11 weeks of iron supplementation. The important findings indicate that 10 weeks after cessation of supplementation (T21) the benefits of iron supplementation in ITG completely disappeared at T29. Therefore, our data suggests that during a competitive season any advantage of iron status due to supplementation will dissipate in elite female volleyballers within 18 weeks of supplement cessation. To the best of our knowledge, this is the first study to analyze changes in iron stores in elite female athletes during a season following cessation of iron supplementation. Previous data have indicated that iron stores are diminished without supplementation over 11 weeks in female volleyballers [4], but are sustained during the season with supplementation with 105 mg/day elementary iron [9]. Presently, iron stores were depleted almost completely to baseline levels in ITG within 10 weeks of supplement cessation, and were completely dissipated after 18 weeks total. Thus, it seems that supplementation should be sustained throughout an entire season to maintain the benefits.

A curious observation was the significant increase of FER levels in CG from T11 to T18 even though the same diet and training occurred in CG and ITG. Due to the significant increase in FER, there was the number of players with iron deficiency also decreased from T11 to T18. While the increase in FER could be due to dietary intake, we believe that this is unlikely as all diets were prescribed by a dietitian who considered iron absorption factors. In addition, there were no differences between groups for intake of any macro or micronutrient. Therefore, we speculate that the FER increase during this period could be due to a few mechanisms. One possible explanation is that low iron stores cause a decrease in hepcidin, which results in increased iron uptake at the intestinal level [25]. Additionally, in the original trial [9], subjects in CG experienced a decrease in strength during the season, which may have generated pro-inflammatory/inflammatory cytokines and acute phase proteins which would interfere with the supply of iron. Ultimately, this could result in changes in iron metabolism and negatively affect iron availability for erythropoiesis [26].

Interestingly, even though strength levels in ITG were maintained from T11 to T29, there were significantly greater percentage changes in strength in CG vs. ITG over the 18 weeks in clean and jerk, power clean, and TMS. One possible explanation is the accompanying greater change in FER and Hb levels in CG vs. ITG from T11 to T21, and the significant percentage increase in FER from T11 to T29 in CG, but no significant change in ITG. In further support, we observed a significant relationship between ∆FER and ∆TMS, which is one of the parameters that increased to a greater extent in CG than ITG. A second explanation of greater strength changes at T29 in favor of CG is that the initial 11 weeks of supplementation were not placebo controlled. In other words, expectation of results in response to a treatment can affect the outcome; therefore, it is possible that the athletes in ITG did not improve strength due to psychological concerns that supplementation had ceased.

It is important to note that excessive iron supplementation can lead to toxicity [27] and cause harm [13]. During both the initial 11-week supplementation period [9] and the present 18-week follow-up athletes met the RDA for iron and there was no significant difference between groups (*p* > 0.05) for total caloric intake. As a result, it does seem that iron supplementation can be used to prevent a decline in iron stores while avoiding toxicity in female athletes even when the iron RDA is met and caloric intake is sufficient. Furthermore, from T11 to T29, two players in ITG shifted iron categorization from functional iron deficiency to absolute deficiency, while CG did not have any players experience a negative change in iron categorization. Therefore, while iron supplementation should only be carried out after a blood panel is taken; it does seem to be safe when consistent and demanding training is occurring in female athletes.

As previously mentioned, a possible limitation is that the primary trial was not placebo-controlled, which could have affected athletes’ performance expectations during both the supplementation period and once supplementation ended. However, to counter this notion we did observe a relationship from T21 to T29 between ∆FER and ∆Hb with certain strength tests. Furthermore, it is possible that a higher dosage than the 325 mg/day of ferrous sulphate in the first 11 weeks or maintaining supplementation for a longer period would have led to a sustained benefit in iron storage through T29.

## 5. Conclusions

In summary, the improved iron stores and iron status categorization that were achieved due to supplementation at T11 were not maintained during the following 18 weeks when supplementation was stopped. Furthermore, strength levels were not different between groups 18 weeks after supplementation, despite ITG having greater strength during the last week of supplementation (T11). Practically, female volleyball players may be at risk for ID when engaged in the demands of a competitive season. Therefore, dietary intake should be tracked to ensure that iron RDA requirements are met, however, our data suggest that this is not enough to prevent a decline in iron stores and performance, and blood panels should be assessed to determine if iron supplementation is necessary. Ultimately, if iron supplementation is used to prevent ID and improve performance during a competitive season in elite female volley ballers, supplementation should be considered.

## Figures and Tables

**Table 1:** Iron Categorization in control group (CG; *n* = 11) and iron treatment group (ITG; *n* = 11) at finish of iron treatment (T11) and at the finish of season (T29).

	Adecuate Iron Stores	Funtional Iron Deficency	Absolute Iron Deficency	Anemia	*p*
CG					
T11	0 (0)	7 (63.6)	4 (36.4)	0 (0)	0.157
T29	1 (9.1)	7 (63.6)	3 (27.3)	0 (0)	
ITG					
T11	2 (18.2)	7 (63.6)	2 (18.2)	0 (0)	0.046
T29	1 (9.1)	6 (54.5)	4 (36.4)	0 (0)	

Note: Data expressed in frequency (%).

**Table 2:** Regression multivariate analysis with strength tests as the dependent variable.

	Unstandardized Coefficients		Standardized Coefficients	*t*	Sig.	*R*^2^ Adjust
	B	Std. Error	Beta			
∆ Half-squat						
(Constant)	−8.588	3.361		−2.555	0.031	
∆TRF	−0.967	0.332	−0.696	−2.910	0.017	0.427
∆ Pull Over						
(Constant)	3.539	2.470		1.433	0.186	
∆FER	0.123	0.032	0.788	3.839	0.004	0.580
∆ Total Mean Strength						
(Constant)	−2.735	1.481		−1.847	0.098	
∆TRF	−0.501	0.148	−0.578	−3.387	0.008	0.689
∆FER	0.048	0.015	0.556	3.259	0.010	

**Table 3:** Energy, macro and micronutrients intake in control group (CG) and iron treatment group (ITG) during follow up study.

	CON	ITG	*p*	RDA *
Energy (kcal)	2744.5 ± 21.6	2792.4 ± 34.4	0.279	
Total Carbohydrates (g)	288.6 ± 24.2	306.22 ± 22.0	0.105	
Total Proteins (g)	134.6 ± 14.2	138.2 ± 19.3	0.648	
Animal Proteins (g)	91.16 ± 15.6	95.2 ± 19.0	0.618	
Vegetable Proteins (g)	43.6 ± 6.1	43.2 ± 3.5	0.856	
Total Fats (g)	111.4 ± 9.0	108.7 ± 8.8	0.515	
Ca (mg)	1168.5 ± 60.2	1166.7 ± 66.8	0.984	1000
Mg (mg)	541.0 ± 31.3	554.5 ± 17.9	0.699	310
P (mg)	2106.1 ± 45.0	2126.2 ± 84.4	0.846	700
Fe (mg)	22.1 ± 1.5	23.52 ± 1.12	0.454	18
Zn (mg)	14.0 ± 0.3	14.4 ± 0.7	0.605	8
Vitamin A (µg)	1791.3 ± 300.3	1862.9 ± 247.5	0.708	700
Vitamin E (mg)	18.3 ± 1.2	15.5 ± 0.7	0.051	15
Thiamine (mg)	2.52 ± 0.08	2.43 ± 0.14	0.604	1.1
Riboflavin (mg)	2.70 ± 0.06	2.79 ± 0.17	0.678	1.1
Niacin (mg)	37.9 ± 2.2	38.9 ± 2.3	0.749	14
Vitamin B6 (mg)	3.77 ± 0.19	3.82 ± 0.25	0.868	1.3
Folic Acid (µg)	624.8 ± 47.7	621.3 ± 39.9	0.955	400
Vitamin B12 (µg)	9.24 ± 0.99	9.96 ± 2.01	0.767	2.4
Vitamin C (mg)	358.4 ± 47.7	371.4 ± 36.6	0.829	75

* RDA: Recommended Dietary Allowances for women aged 19–30 [16].
